# Factors associated with anemia among Sri Lankan primary school children in rural North Central Province

**DOI:** 10.1186/s12887-017-0841-9

**Published:** 2017-03-27

**Authors:** Gayani Shashikala Amarasinghe, Naotunna Palliya Guruge Chamidri Randika Naottunna, Thilini Chanchala Agampodi, Suneth Buddhika Agampodi

**Affiliations:** Maternal and Child Health Research Unit, Department of Community Medicine, Faculty of Medicine and Allied Sciences, Rajarata University of Sri Lanka, Saliyapura, 50008 Sri Lanka

**Keywords:** Anemia, Primary school children, Sri Lanka, Anuradhapura, Polonnaruwa, North Central Province, Rural

## Abstract

**Background:**

Despite interventions, childhood anemia is still a major public health problem in low and middle income countries. Purpose of the present study is to determine factors associated with anemia among rural primary school children in Sri Lanka, a country undergoing rapid socioeconomic changes.

**Methods:**

Multi stage cluster sampling was used to select 100 rural schools in NCP and a maximum of 50 children aged 60–131 months were enrolled from each school. Self-administered questionnaires were sent to parents. Anthropometric measurements and blood samples were obtained by trained investigators. Blood reports were analyzed in a commercial laboratory with external quality control measures.

**Results:**

Total of 4412 children were included in the analysis. A Multiple regression was performed for hemoglobin. Only 4.2% of the change in hemoglobin could be explained by the model. District (*p* > 0.001), age (*p* > 0.001), timing of warm treatment(*p* = 0.026) and BMI for age (*p* = 0.002) uniquely contributed 1.12%, 1.19%, 0.13% and 0.26% to change in hemoglobin level respectively whereas, sex, monthly family income and frequency of meat and green leaf consumption didn’t contribute significantly.

Peripheral blood film analyses were available for 146 anemic children. Blood film was reported as normal in 19.9% while evidence of iron deficiency (18.5%), early iron deficiency (32.5%) and thalassemia trait with iron deficiency (29.5%) were reported in the rest.

Serum ferritin level was available for 417 children with hemoglobin less than 12 g/dl. Mean ferritin level was 63.7 microgram/l. Only 0.5% had depleted iron stores. A multiple regression was performed for serum ferritin and R^2^ was 0.123 (*p* < 0.001). Area under the curve for serum ferritin and anemia was 0.436.

**Conclusion:**

Anemia among rural primary school children in NCP cannot be well explained by routinely assessed socioeconomic characteristics which mainly provide clues to access for food. Commonly used anemia related investigations have low validity in detecting and explaining anemia in this population. Since behavioral factors have been shown to affect nutrition of younger children in Sri Lanka, studying weather behaviors are related to anemia in primary school children is important. Possible etiologies including but not limited to nutritional deficiencies need to be studied further.

## Background

Anemia is a condition in which the number of red blood cells or their oxygen-carrying capacity is insufficient to meet physiologic needs [1]. Anemia affects around 305 million school age children globally [2]. It is estimated that about 50% of global anemia is due to iron deficiency [3].

Anemia has a variety of adverse health outcomes including mortality, depending on severity and characteristics of affected population. Anemia during infancy and childhood can impair immunity [4–7]. Anemia, specially iron deficiency anemia affects cognitive performances [8] and school performances in children [9–11]. It is also suggested that anemia increases the heavy metal absorption in children [12]. It affects the exercise tolerance [13] and hormonal regulation in adults [14].

Anemia has been reported as a major child health issue among Sri Lankan children since early 19^th^ century. Early, extensive studies on childhood anemia in Sri Lanka identified nutritional anemia as the main etiology, while thalassemia and malaria were also reported as additional causes [15–22]. Recent studies on anemia among Sri Lankan children are mainly focused on preschoolers and teenagers. These studies show a varying degree of anemia prevalence ranging from 15.1% [23] to 33% [24] among children under 5 years of age and 11.1% [25] to 54.8% [26] among teenagers.

Anemia prevalence among Sri Lankan primary school children has been reported as 58% by National health and nutrition Survey (1995) [27], 84% in Monaragala (1997) [28] and 52.7% in a hospital based study in Colombo district (2003) [29]. In all these studies, hemoglobin less than 12 g/dl has been considered as the cut off. In a national wide survey in 2002, 12.1% of 9–10 years old children were found to be anemic [30]. Though studies are available on infants, preschool children and teenagers, resent studies on distribution and determinants of anemia among primary school children in Sri Lanka are limited. Moreover, health inequalities within the country have not been considered in most of the studies.

The national family health programme in Sri Lanka has implemented several anemia prevention interventions targeting different stages of life cycle. For an example, school medical inspection (SMI) programme is conducted nationally in all the schools. It includes providing iron and folate supplements, deworming treatment, and health education to students [31, 32]. National wide awareness programmes are conducted to prevent Thalassemia [33]. Even though malaria has been endemic in the past, indigenous cases of malaria have not been reported in Sri Lanka since 2012 [34]. With health interventions and rapid socioeconomic changes taking place in the country, the epidemiology of childhood anemia can be expected to be changed significantly, thus requiring further investigations on underlying causes.

We previously reported that anemia prevalence among rural primary school children in North Central Province (NCP), Sri Lanka was 17.1% and mean hemoglobin level is 12.2 g/dl (SD = 0.89) [35]. Hemoglobin level less than 11.5 g/dl was used as the cut off; as recommended by the WHO for 5–11 years old children living at altitudes of less than 1000 m [36]. Initial analysis has shown that the prevalence declined with age in both sexes [35]. Purpose of the present paper is to determine modifiable factors associated with childhood anemia in NCP in order to plan evidence based public health interventions.

### Methods

This study was a part of a large study on nutritional status of school children in NCP. Detailed methodology of the original study is published elsewhere [35]. A summary is presented here.

The two districts in NCP; Anuradhapura and Polonnaruwa have estimated midyear populations of 883 000 and 415 000 respectively [37]. In year 2012/2013 mean household income per month was 36632 Rupees (243 USD) and poverty headcount index (the percentage of population living below the official poverty line) was 7.3% [38]. Out of 802 schools in the province, 701 (with a total student population of 93 243) are considered as rural schools.

Population for this school based cross sectional study was 60–131 months old children, studying in rural schools in the NCP. A multi stage cluster sampling with probability proportionate to the size was used to select 100 rural schools. Maximum of 50 students per each school were enrolled using a simple random sampling technique. Socio-demographic information was obtained from a self-administered questionnaire sent to parents/guardians through school principals prior to sample collection. Response rate was 97.6% (*n* = 4521). Anthropometric measurements were obtained by trained medical graduates and laboratory samples were collected by trained nurses. Investigation reports were obtained from a commercial diagnostic laboratory with external quality control procedures. Further details on study design, including study setting, study population, sampling method, data collection tools and data collection are described in the previous paper [35].

IBM SPSS version 20 was used for data analysis. First, mean hemoglobin levels and anemia prevalence between different groups were compared using independent sample *t* test or one way analysis of variance (ANOVA). Standard multiple regression was performed including hemoglobin as the dependent variable. District (Anuradhapura, Polonnaruwa), Sex and Ethnicity (Sinhala, Muslim) were entered as binary variables. Age, average monthly income of the family, Body Mass Index (BMI), maternal and paternal education (last grade passed at school) and number of children in the family were entered as quantitative variables. When the last worm treatment was given (within less than one month, within one month to three months, within three to six months, within six to twelve months and more than 12 months back) was entered as a categorical variable. Average number of meals containing meat and green leaves per week were binned to quartiles due to considerable number of unrealistic values reported by parents.

Results of peripheral blood film were analyzed using ANOVA.

Another standard multiple regression was performed including serum ferritin as the dependent variable. Independent variables were entered similar to as for hemoglobin except for the omission of district and addition of hemoglobin. Hemoglobin was entered as a quantitative variable.

Preliminary tests were performed for linearity, multicolinearity and normality for both regressions.

## Results

Hemoglobin level was available for 4412 out of 4521 children in the original sample (see Table 1).

**Table 1:** Characteristics of study participants

	Number	Percent
District		
Anuradhapura	2480	56.2
Polonnaruwa	1932	43.8
Sex		
Males	2228	50.8
Females	2168	49.1
Ethnicity		
Sinhala	3704	84.0
Muslim	384	8.7
Tamil	6	0.1
Education division		
Kabathigollawa	713	16.2
Galenbindunuwewa	514	11.7
Anuradhapura	379	8.6
Thabuththegama	502	11.4
Dibulagala	538	12.2
Kekirawa	372	8.4
Higurakgoda	838	19.0
Polonnaruwa	556	12.6
No. of children in the family		
Less than 3	2474	61.2
3 or more	1567	38.7
Average family income per day ($)		
< $1	292	8.1
$1-2	391	10.8
$2-3	535	14.8
$3-4	112	3.1
$4-8	1558	43.0
$8-12	593	16.4
> $12	142	3.9
Father’s education level		
Primary	762	19.2
Up to O/L	2737	69.0
Above O/L	469	11.8
Mother’s education level		
Primary	616	15.3
Up to O/L	2732	67.9
Above O/L	675	16.8
Last worm treatment		
Within 3 months	2824	72.3
3 to 6 months back	893	22.8
Before 6 months	191	4.8
Frequency of meat consumption		
< 1^st^Quartile	1890	48.4
1^st^ to 2^nd^Quartile	953	24.4
2^nd^ to 3^rd^Quartile	476	12.2
> 4^th^ Quartile	584	15.0
Frequency of green leaf consumption		
< 1st Quartile	1671	42.0
1^st^ to 2^nd^ Quartile	838	21.1
2^nd^ to 3^rd^ Quartile	654	16.5
> 4th Quartile	812	20.4

No significant difference in anemia prevalence (Pearson Chi-square 0.819, *p* = 0.365) or mean hemoglobin level (t = -1.042, *p* = 0.297) was observed between males and females. This was true for both Anuradhapura and Polonnaruwa districts individually and among ethnic Sinhalese and Muslims (Table 2).

**Table 2:** Comparison of anemia prevalence and mean hemoglobin concentration between males and females across districts and ethnicities

			Anemia prevalence (%)	Hemoglobin (g/dl)			
				Mean	SD	t value	*p* value
District	Anuradhapura	Male	20.0 (N = 253)	12.07	0.94	-0.802	0.423
		Female	20.2 (N = 245)	12.10	0.86		
	Polonnaruwa	Male	14.4 (N = 139)	12.32	0.88	-0.545	0.586
		Female	11.8 (N = 113)	12.34	0.85		
Ethnicity	Sinhala	Male	18.3 (N = 346)	12.15	0.93	-1.519	0.129
		Female	16.7 (N = 302)	12.20	0.88		
		Total	17.5 (N = 648)	12.18	0.90		
	Muslim	Male	12.0 (N = 22)	12.27	0.78	0.295	0.768
		Female	14.5 (N = 29)	12.25	0.81		
		Total	13.3 (N = 51)	12.26	0.80		

Mean hemoglobin levels were significantly different between Sinhalese and Muslims (t = -1.996, *p* = 0.047) (Table 2).

The education divisions in Polonnaruwa district (Polonnaruwa, Hingurakkgoda and Dimbulagala) had lower prevalence of anemia compared to Anuradhapura divisions. Children from families with less than three children had higher mean hemoglobin level (*p* = 0.016) compared to children from families with three or more children. Mean hemoglobin level increased with increased education level of father (*p* = 0.001) and mother (*p* = 0.005). Mean hemoglobin levels were not significantly different between children with different average family income levels (*p* = 0.194) or across quartiles of parent reported frequency of meat (*p* = 0.559) or green leaf (*p* = 0.864) consumption per week (Table 3).

**Table 3:** Mean hemoglobin level and prevalence of anemia among rural primary school children in North Central Province, Sri Lanka

	Anemia prevalence %	Hemoglobin		
		Mean	SD	Significance
Educational division				
Kabathigollawa	23.1(N = 165)	12.02	0.95	F = 16.12, p = 0.001
Galenbindunuwewa	21.4(N = 110)	12.03	0.92	
Anuradhapura	17.7(N = 67)	12.08	0.84	
Thabuththegama	20.5(N = 103)	12.12	0.90	
Dibulagala	14.3(N = 77)	12.20	0.81	
Kekirawa	14.5(N = 54)	12.22	0.83	
Higurakgoda	13.0(N = 109)	12.34	0.85	
Polonnaruwa	12.2(N = 68)	12.42	0.92	
No. of children in the family				
Less than 3	16.3(N = 403)	12.21	0.88	t = 2.413, p = 0.016
3 or more	18.3(N = 286)	12.14	0.92	
Average family income per day ($)				
< $1	20.2(N = 59)	12.15	0.89	F = 1.444, p = 0.194
$1-2	16.1(N = 63)	12.24	0.94	
$2-3	16.8(N = 90)	12.17	0.92	
$3-4	11.6(N = 13)	12.25	0.88	
$4-8	17.8(N = 278)	12.15	0.88	
$8-12	15.7(N = 93)	12.23	0.88	
> $12	19.0(N = 27)	12.27	0.84	
Father’s education level				
Primary	16.5 (N = 126)	12.12	0.87	F = 7.236, p = 0.001
Up to O/L	17.6 (N = 483)	12.19	0.90	
Above O/L	14.3 (N = 67)	12.32	0.88	
Mother’s education level				
Primary	17.4 (N = 107)	12.13	0.88	F = 5.330, p = 0.005
Up to O/L	17.5 (N = 477)	12.17	0.91	
Above O/L	15.4 (N = 104)	12.28	0.85	
Last worm treatment				
Within 3 months	15.9 (N = 449)	12.21	0.87	F = 1.087, p = 0.337
3 to 6 months back	19.0 (N = 170)	12.16	0.92	
Before 6 months	15.7 (N = 30)	12.17	0.86	
Frequency of meat consumption				
< 1^st^ Quartile	17.5 (N = 331)	12.18	0.91	F = 0.689, p = 0.559
1^st^ to 2^nd^ Quartile	15.6 (N = 149)	12.21	0.88	
2^nd^ to 3^rd^ Quartile	18.3 (N = 87)	12.14	0.89	
> 4^th^ Quartile	18.2 (N = 106)	12.19	0.88	
Frequency of green leaf consumption				
< 1st Quartile	16.8 (N = 281)	12.20	0.91	F = 0.247, p = 0.864
1^st^ to 2^nd^ Quartile	17.1 (N = 143)	12.17	0.96	
2^nd^ to 3^rd^ Quartile	17.4 (N = 114)	12.17	0.85	
> 4th Quartile	17.4 (N = 141)	12.18	0.84	

No significant difference in mean hemoglobin level was observed between groups who had received worm treatment within last three months, within three to six months or more than six months back (*p* = 0.337)(Table 3). However, when groups who had received worm treatment less than and more than one month back were compared, mean hemoglobin level was higher in the former group (t = 3.413, *p* = 0.001).

There was a week positive correlation between hemoglobin and weight for age (*r* = .079, *p* < 0.001), height for age (*r* = 0.041, *p* = 0.007) and BMI for age (*r* = 0.062, *p* < 0.01).

A standard multiple regression was performed with hemoglobin as the dependent variable. (R^2^ = 0.042, *p* < 0.001) (Table 4). Preliminary tests were conducted for linearity, multicolinearity, and normality. Maternal education showed a strong colinearity with paternal education (*r* =0 .821, *p* < 0.001) but both variables were allowed in the model since influence on each other practically is not likely. None of the variables had tolerance (1-R) less than 0.1 and Variance Influencing Factor was also less than 10 in all variables.

**Table 4:** Multiple regression for hemoglobin in rural primary school children in the NCP, Sri Lanka

Variable	r^1^	p^1^	N	Beta	p^2^	r^2^
District	0.134	0.000	4412	0.114	0.000	0.106
Sex	0.016	0.149	4396	0.009	0.607	0.009
Ethnicity	0.028	0.036	4088	0.023	0.197	0.022
Age (days)	0.139	0.000	4412	0.140	0.000	0.139
Father's education	0.056	0.000	3968	0.037	0.202	0.021
Mother's education	0.049	0.001	4023	-0.016	0.586	-0.009
Average monthly income of family	0.013	0.217	3623	0.010	0.548	0.010
Number of children in family	-0.015	0.171	4041	-0.028	0.113	-0.026
Frequency of green leaf consumption	-0.008	0.307	3975	-0.019	0.281	-0.018
Frequency of meat consumption	0.001	0.485	3903	0.011	0.542	0.010
Worm treatment	-0.041	0.006	3908	-0.039	0.026	-0.037
BMI for age	0.062	0.000	4328	0.052	0.002	0.051

r- Pearson correlation, p^1^- Significance of Pearson correlation, *N*- Number, Beta - Standardized Coefficient, p^2^ - Significance of standardized coefficient, r^2 –^Part Correlation

District, age, timing of worm treatment and BMI for age uniquely contributed 1.12%, 1.19%, 0.13% and 0.26% respectively to changes in hemoglobin level.

Peripheral blood film analyses were available for 146 children with anemia (Table 5).

**Table 5:** Results of peripheral blood film analyses of anemic rural primary school children in NCP

Reporting on peripheral blood film	Mild anemia^a^ (%)	Moderate anemia^b^ (%)	Total* (%)	Hemoglobin (g/dl)		
				Mean	SD	F (p)
Normal	28 (36.8%)	1 (1.4%)	29 (19.9%)	11.21	0.17	26.2 (0.001)
Iron deficiency anemia	6 (7.9%)	21 (30.0%)	27 (18.5%)	10.37	0.80	
Early iron deficiency	32 (42.1%)	15 (21.4%)	47 (32.2%)	11.08	0.26	
Thalassemia trait with iron deficiency	10 (13.2%)	33 (47.1%)	43 (29.5%)	10.42	0.60	

*No severe anemia cases (hemoglobin less than 8 g/dl) were present

^a^hemoglobin 11-11.4 g/dl [36]

^b^hemoglobin 8-10.9 g/dl [36]

When Post-Hoc tests were performed, groups having iron deficiency anemia (IDA) and thalassemia trait with iron deficiency (TT with ID) were having significantly lower mean hemoglobin levels than those who were reported as normal or having early iron deficiency (EID) (*p* < 0.0001 in all instances). There were no significant differences in mean hemoglobin level between children with IDA and TT with ID (*p* = 0.98) and between children with normal peripheral blood film analysis and EID (*p* = 0.66). Mean serum ferritin levels were not different between these groups (*F* = 1.5, *p* = 0.22).

Serum ferritin reports were available for 417 out of 674 children in Polonnaruwa who had hemoglobin less than 12 g/dl. Out of them, 199 were anemic. Mean S. Ferritin level was 63.7 microgram/l (SD = 37.6). Only 0.5% (*N* = 2) had depleted iron stores (WHO recommended cutoff of Serum ferritin less than 15 microgram/l was used [39]).

A standard multiple regression was performed with S. ferritin when hemoglobin was less than 12 g/dl as the dependent variable (Table 6). R^2^ was 0.123 (*p* < 0.001). Only hemoglobin, average monthly income of family, sex, frequency of meat consumption and number of children in the family were contributing significantly to changes in serum ferritin.

**Table 6:** Multiple linear regression for ferritin in rural primary school children in Polonnaruwa district who have hemoglobin levels less than 12 g/dl

	r^1^	p^1^	N	Beta	p^2^	r^2^
Hemoglobin	-0.241	0.000	417	-0.248	0.000	-0.244
Average monthly income of family	-0.131	0.006	359	-0.136	0.008	-0.134
Frequency of meat consumption	0.114	0.013	384	0.134	0.014	0.125
Sex	-0.109	0.013	413	-0.108	0.033	-0.108
Mother's education	-0.080	0.057	397	-0.081	0.362	-0.046
Father's education	-0.064	0.103	389	0.033	0.710	0.019
Age (days)	0.065	0.092	417	0.097	0.059	0.095
Frequency of green leaf consumption	0.037	0.237	382	-0.003	0.961	-0.002
BMI for age	-0.003	0.477	403	0.025	0.627	0.024
Ethnicity	-0.002	0.488	405	-0.029	0.593	-0.027
Number of children in the family	0.098	0.025	402	0.082	0.120	0.078
Timing of warm treatment	0.028	0.289	396	0.006	0.906	0.006

r^1^- Pearson correlation, p^1^- Significance of Pearson correlation, N- Number, Beta - Standardized Coefficient, p^2^ - Significance of standardized coefficient, r^2 –^Part correlation

**Fig. 1:**
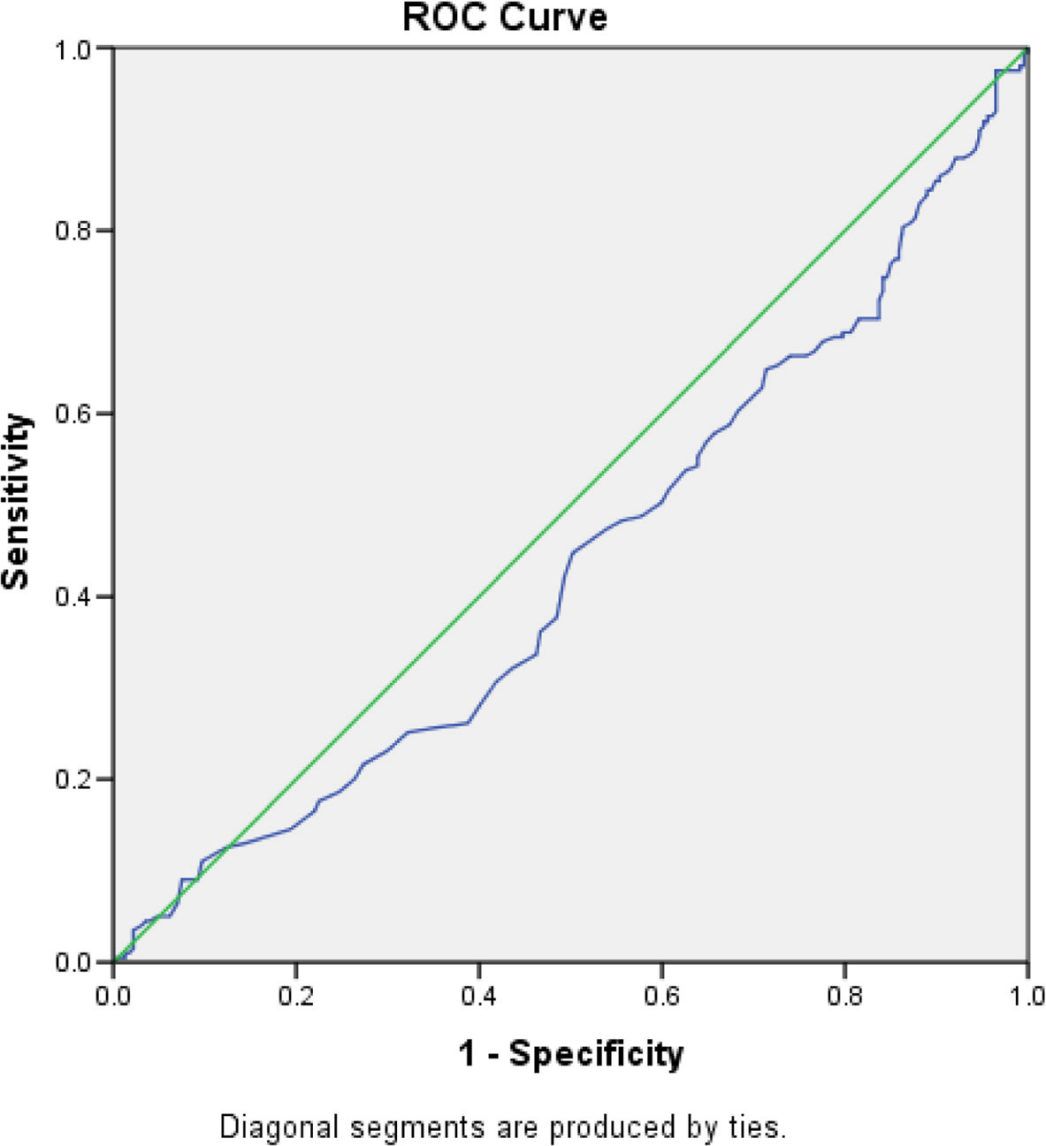
Receiver Operated Curve for serum ferritin level and anemia in rural primary school children in NCP with hemoglobin less than 12 g/dl

When Receiver operated curve (ROC) was drawn for serum ferritin and anemia area under the curve was 0.436 (Fig. 1).

## Discussion

This is the largest reported study available in the literature on childhood anemia in a single province in Sri Lanka. The study design enabled us to produce generalizable data on this rural population as a case study on anemia among primary school children in rural Sri Lanka. This study may be representing the changes in determinants of childhood anemia in low and middle income countries undergoing rapid epidemiological transition. Further, the study provides important clues about the impact of health inequalities within the country and the rural population itself on childhood nutrition.

A significant difference was observed between the two districts showing subnational variations in anemia prevalence. This difference in hemoglobin level among children in two districts was an unexpected finding and it was independent from other factors such as differences in income, ethnic distribution, and parental education between the two districts. According to the demographic and health survey (DHS) 2006/2007 the lowest prevalence of anemia among under five children in Sri Lanka was reported in Polonnaruwa (15.2%) while a higher prevalence (31.0%) was reported in Anuradhapura [24]. Since the current study was conducted in 2014, population from which this study sample was driven may considerably overlaps with the 6 to 59 months old population studied in DHS 2007. However, according to national nutrient and micronutrient survey (NNMS) 2012, anemia prevalence in 6–59 months old children was lower in Anuradhapura (16.2%) than in Polonnaruwa (21.5%) [23]. The observed difference in anemia prevalence between the two districts in two time points may be due to a cohort effect which needs further investigation.

Sex doesn’t contribute to differences in hemoglobin level in this study population. No difference in mean hemoglobin level was observed among males and females, in either districts or among different ethnicities (Sinhala and Muslim). Minimal discrimination against female child, as compared to some cultures in the region could be a major contributory factor for this observation. However according to previous studies, males were having higher anemia prevalence among 6–59 months old children [23] whereas females were having the higher prevalence among teenagers; specially after menarche [26].

An association between deworming and anemia has been described in Sri Lanka. A study in 2002 showed that prevalence of intestinal helminthes among a national representative sample of Sri Lankan primary school children was 6.9% [30]. Worm treatment is provided to students during SMI programme and based on routine data, SMI coverage was 85.7% in Anuradhapura and 80.9% in Polonnaruwa districts [40]. In current study 95.4% of children had received worm treatment within last six months. Considering that shorter intervals after deworming was associated with better hemoglobin levels in this study, paying attention to control of transmission of intestinal helminthes can be viewed as important as periodical deworming.

In contrast to findings from most of the studies on childhood anemia in developing countries, economic status was not correlated with hemoglobin level in this population. Even in Sri Lanka, the NNMS has reported an association between monthly income and anemia in 6–59 months old children [23]. However, our study population is more homogenous compared to that of the national survey and therefore can be assumed to provide more generalizable data on rural community. Access to food was not observed as a significant contributory factor in this study. This statistical model could explain only 4.2% of the change in hemoglobin level of 60–131 months old children in rural Sri Lanka. Therefor hemoglobin has to be affected by factors other than socio, economic and demographic characteristics analyzed in this study. Evidence are appearing that certain behaviors of caregivers affect the nutrition state of infants and young children in Sri Lanka [41–44]. Behavioral factors may be affecting hemoglobin level of primary school children as well.

However, this study provides limited information on food consumption patterns and even less so on the determinants of such behaviors. A more structured assessment of them needs to be carried out in further studies in order to find out and modify factors contributing to anemia among primary school children in Sri Lanka.

Observations on results of peripheral blood film analysis raise questions on validity of it as an investigation supplementing the diagnosis and explanation of anemia. They are heavily used in day today clinical practice for this purpose and if there are issues on accuracy it might affect the accuracy of clinical decisions. These observations are also suggestive that it may be worth to carry out further research to establish whether or not an alternative cutoff hemoglobin level is more suitable to define anemia in this population.

Iron deficiency is considered as the commonest cause of anemia among children specially in low and middle income countries [3]. Epidemiological studies and interventions in these settings usually focus on determinants of iron deficiency or broadly, nutritional anemias. In this study, only two (0.5%) children out of 417 had depleted iron stores. It seems to be an underestimation, compared to the prevalence of anemia. Previous studies also have reported that iron deficiency may not be the leading cause of anemia in rural Sri Lanka [28]. Prevalence of other micronutrient deficiencies (e.g. Folate) have been described among Sri Lankan children which may be a cause for anemia in this sample also [26, 45]. However, higher prevalence of iron deficiency was predicted by peripheral blood film analysis of the current sample. Higher prevalence of depleted iron stores in preschoolers and adolescents have been identified in other parts of the country [26, 45, 46]. Serum Ferritin depends on other factors such as presence of haemoglobinopathies [47] and inflammation [48]. According to peripheral blood film analyses 29.5% anemic children were having thalassemia trait with iron deficiency. Some studies even argue that serum ferritin should not be used alone to determine iron status [49]. Faulty estimation of S. Ferritin can occur due to variety of reasons also [50]. Area under the curve being 0.436 also supports that serum ferritin is a poor test with regards to predicting anemia in this study sample.

Conclusive evidence on etiological basis of anemia could not be obtained from peripheral blood film analysis or serum ferritin estimations. Therefore, estimation of iron stores and identification of hemoglobinopathies need to be carried out using more reliable tests.

As the study was based in rural primary schools, results may not be generalizable to children attending other schools with higher resources and access. Even though small in percentage (net school enrolment is 99.8% in rural areas [51]), children who are not attending school are also left out in this study. Data about food consumption patterns were not obtained using validated food frequency questionnaires. A complete picture about population iron stores cannot be derived as serum ferritin estimates were available only for children from Polonnaruwa who had hemoglobin less than 12 g/dl. Comparing serum ferritin levels in children from the two districts could have provided important clues to the cause of cohort effect resulting in lower anemia prevalence in Polonnaruwa. If inflammatory conditions at the time of sample collection could be identified by testing for inflammatory markers interpretation of ferritin levels would have been more accurate.

**Table:** 

Unique contribution from this study This is the largest reported study available in the literature on childhood anemia in a single province in Sri Lanka. This is a school based study investigating into prevalence and determinants of anemia among rural primary school children, which has not been studied thoroughly in the past. The study provides important clues about how health inequalities within the country and the rural population itself affect childhood nutrition. This study may be representing the changes in determinants of childhood anemia in low and middle income countries undergoing rapid epidemiological transition	

## Conclusion

Anemia is still a public health problem among primary school children in NCP. It cannot be well explained by routinely assessed demographic or socio economic characteristics which mainly give clues to access for food. Commonly used anemia related investigations are having a low validity in detecting and explaining anemia in this population. Since evidence on behavioral factors affecting the nutrition of infants and young children are emerging in Sri Lanka, studying the effect of behaviors on anemia among primary school children will be important. Possible etiologies including but not limited to nutritional deficiencies needs to be studied further.

## Abbreviations

ANOVA: Analysis of variance
BMI: Body mass index
DHS: Demographic and health survey
EID: Early iron deficiency
IDA: Iron deficiency anemia
NCP: North Central Province
NNMS: National nutrient and micronutrient survey
ROC: Receiver operated curve
SMI: School medical inspection
TT: Thalassemia trait
WHO: World Health Organization
